# The effect of frontal trauma on the edentulous mandible with four different interforaminal implant-prosthodontic anchoring configurations. A 3D finite element analysis

**DOI:** 10.1186/s40001-023-01580-y

**Published:** 2023-12-19

**Authors:** Stefan Krennmair, Michael Malek, Raphael Stehrer, Philip Stähler, Sven Otto, Lukas Postl

**Affiliations:** 1 Medical Faculty, Johannes Kepler University Linz, Altenberger Strasse 69, 4040 Linz, Austria; 2 Department of Oral and Maxillofacial Surgery, Kepler University Hospital, Johannes Kepler University, Krankenhausstrasse 7a, Linz, Austria; 3 NumBioLab, Ludwig-Maximilians University of Munich, Munich, Germany; 4 Department of Oral and Maxillofacial Surgery, Ludwig-Maximilians-University Munich, Lindwurmstrasse 2a, 80337 Munich, Germany

## Abstract

**Purpose:**

The present three-dimensional (3D) finite element analysis (FEA) was aimed to assess the biomechanical effects and fracture risks of four different interforaminal implant-prosthodontic anchoring configurations exposed to frontal trauma.

**Material and methods:**

A symphyseal frontal trauma of 1 MPa was applied to four dental implant models with different configurations (two unsplinted interforaminal implants [2IF-U], two splinted interforaminal implants [2IF-S], four unsplinted interforaminal implants[ 4IF-U], four splinted interforaminal implants [4IF-S]. By using a 3D-FEA analysis the effective cortical bone stress values were evaluated in four defined regions of interest (ROI) (ROI 1: symphyseal area; ROI 2: preforaminal area; ROI 3: mental foraminal area; and ROI 4: condylar neck) followed by a subsequent intermodel comparison.

**Results:**

In all models the frontal traumatic force application revealed the highest stress values in the condylar neck region. In both models with a four-implant configuration (4IF-U, 4IF-S), the stress values in the median mandibular body (ROI 1) and in the condylar neck region (ROI 4) were significantly reduced (P <0.01) compared with the two-implant models (2IF-U, 2IF-S). However, in ROI 1, the model with four splinted implants (4IF-S) showed significantly (P < 0.01) reduced stress values compared to the unsplinted model (4IF-U). In addition, all models showed increased stress patterns in the area adjacent to the posterior implants, which is represented by increased stress values for both 2IF-U and 2IF-S in the preforaminal area (ROI 3) and for the four implant-based models (4IF-U, 4IF-S) in the mental foraminal area.

**Conclusion:**

The configuration of four splinted interforaminal implants showed the most beneficial distribution of stress pattern representing reduced stress distribution and associated reduced fracture risk in anterior symphysis, condylar neck and preforaminal region.

## Introduction

According to the findings of numerous consensus statements, meta-analyses and systematic reviews the use of dental implants has emerged as a well‐accepted treatment modality for oral rehabilitation of edentulism [1–4]. Regardless of implant number, placement timing and procedures performed as well as the anchoring mechanism and the characteristics of the implant-prosthodontic anchoring used, patient satisfaction and comfort has increased significantly compared to conventional complete dentures [4–7].

In an increasingly ageing population, the associated and growing number of elderly patients requiring appropriate treatment of edentulism continues to gain importance [8–10]. In addition, the elderly population has also been shown to be highly physically active suggesting that this active and agile group of elderly patients may be increasingly exposed to the risk of physical maxillofacial trauma [11–13]. Within the field of traumatic maxillofacial lesions mandibular fractures represent the most common facial injuries predominantly related to accidents, violence and falls [14–16]. Moreover, epidemiologic studies have also reported fractures of the atrophic edentulous mandible occurring on account of reduced vascularity and decreased blood flow resulting in atrophy and bone weakening [17, 18].

Because the clinical use of dental implants is considered to increase as a result of significant implant‐prosthodontic advancements, oral and/or maxillofacial surgeons will also be faced with maxillofacial trauma in patients previously treated with dental implants [19–21]. Therefore, the aged population with previous implant-prosthodontic treatment suffering traumatic falls and/or injuries will represent a novel class of maxillofacial trauma patients [12, 17, 22]. Similar to traumatic events in patients containing osteosynthesis material dental implants are seen to alter the biomechanical bone behavior when exposed to traumatic forces [20, 23, 24].

However, only rare information is available on the evaluation of traumatic effects in patients with preceding dental implant treatment. In previous experimental studies, Kan et al. and Ayali and Bilginaylar analyzed two unsplinted implants exposed to traumatic situations using finite element analysis (FEA). Based on their findings, a more beneficial stress modulation was assumingly found for two implants placed in the lateral incisor region than for those placed in the canine region when frontal trauma was present [20, 21]. In an additional experimental study comparing the edentulous mandible without and with four interforaminal implants exposed to frontal trauma it was demonstrated that regardless of splinting or lack of splinting force absorption or transmission may shift the predominant fracture risk factor from the condylar neck to the corpus mandibulae [23].

For the oral rehabilitation of mandibular edentulism two or four interforaminal implants either with splinted bar suprastructure or unsplinted single attachments have been frequently used as a standard implant prosthodontic treatment procedure [7, 25–27]. According to the findings of the McGill consensus form two dental implants—splinted or non-splinted- supporting mandibular prosthesis are described as a minimum number for adequate denture stabilization and retention in the treatment of the completely edentulous mandible [27]. However, additional studies have demonstrated that the use of four implants for denture anchoring provides more rigid attachment by wide-ranging load distribution and reduced rotation than the use of two implants [28, 29]. Moreover, due to the stable anchoring design significantly higher quality of life outcome for patients has been reported for treatment concepts with four-implant splinted bar attachments [26].

In the following study two and four implant bar-connected implants with a round bar design were selected as favorable and most frequently used splinted implant configuration. Additionally, two and four single attachment configurations representing unsplinted anchoring comparing groups were included. Although these four implant-denture anchoring configurations are widely used, there is a lack of information concerning assessment and direct comparisons of traumatic response in situations of frontal trauma exposure. Based on previous literature and considering the lack of clinical data available, this topic of interest might be analyzed using finite element analysis (FEA). The use of FEA represents an appropriate and widely accepted non-invasive method providing valuable reproducible results for estimating various parameters of the complex biomechanics in the oral rehabilitation of mandibular edentulism and behavior of the mandible [20, 21, 32, 33].

The primary aim of this experimental 3-dimensional (3D) FEA study was to evaluate the biomechanical effects of two and four interforaminal implants either in a splinted or unsplinted form under a frontal facial trauma setting. As a secondary objective, the four different implant-denture anchoring configurations evaluated were compared for identifying the configuration form with the most beneficial stress pattern under simulated frontal trauma application.

## Material and methods

### Data acquisition

**Fig.1:**
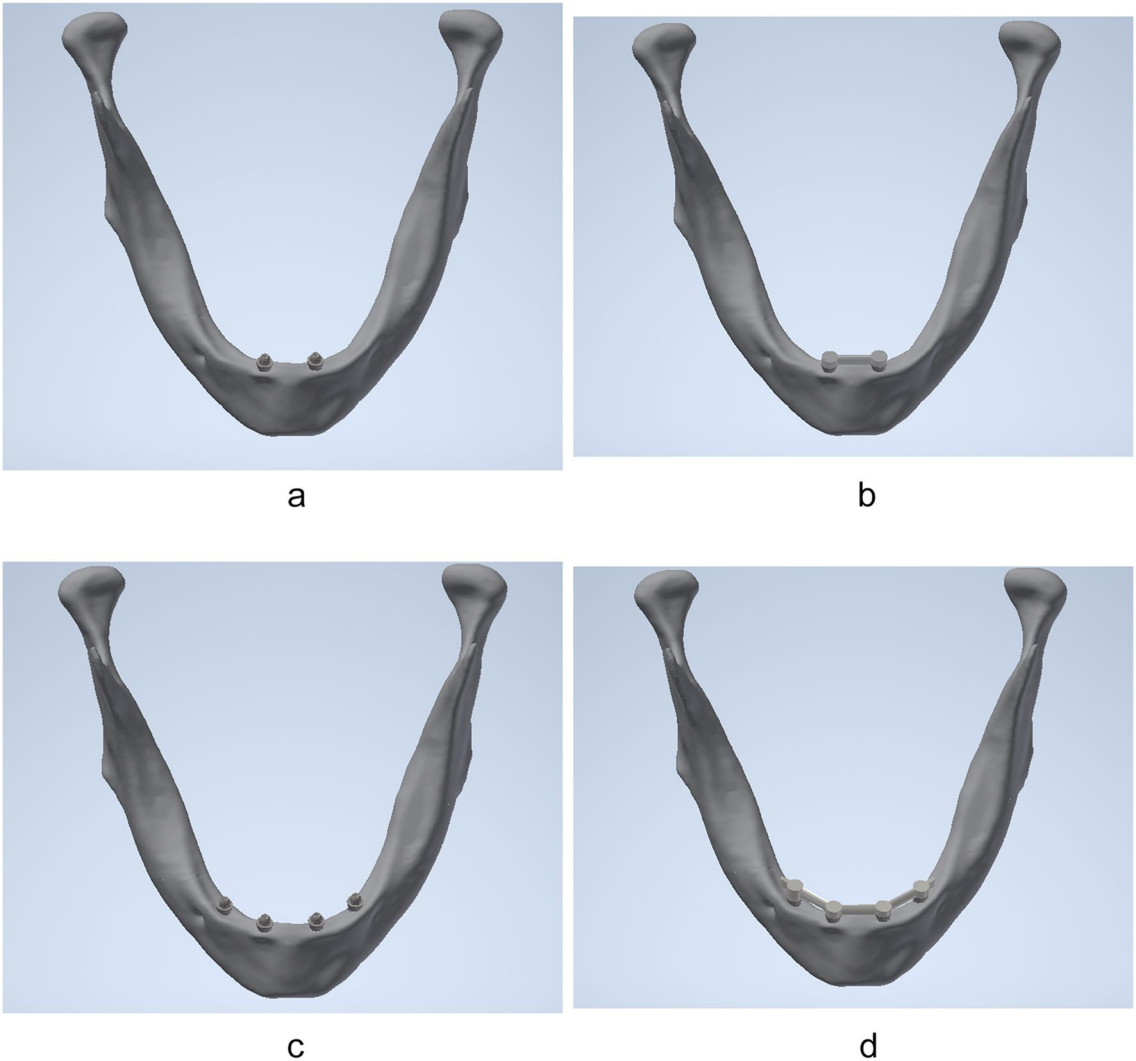
3-D edentulous mandible model with **a** two unsplinted interforaminal implants, **b** two splinted interforaminal implants, **c** four unsplinted interforaminal implants, **d** four splinted interforaminal implants.

A scanned cone-beam computed tomography (CBCT) of a completely edentulous mandible of a 68-year-old male patient was used as the morphological base for the FEM models (ProMax, Planmeca, Helsinki, Finland). The selection of the image data as the anatomical template was based on the patient's medical record status with age-appropriate health and bone status, representing no morphological and mineralization variabilities. The image data with pixel conditions of 651 × 651, at 96 kV, and with increment slices of 0.2 mm in thickness was then converted to DICOM format. Using established image processing software (Amira) the acquisition of cortical and cancellous mandibular bone architecture data was achieved by semi-automatic segmentation of coronary CT layers. The reticulation of point clouds (Delauney-triangulation) to three-dimensional polygon meshes produced morphologically identical sub-models of the cortical and cancellous mandible (Fig. 1).

### CAD modeling

The generated rough polygon meshes could be converted as an DXF (drawing exchange format) to the reverse-engineering software Geomagic Wrap (Geomagic Studio, Rock Hill, SC) in order to generate a smooth computer-aided design (CAD) model of the mandible [34, 35, 35]. The design of all constructible elements of the model, such as implants, abutments, and the superstructure, could be performed virtually using established CAD tools in Inventor software (Autodesk, Munich, Germany) [23, 23, 34]. Dental implants with the dimensions of a Camlog Screw Line Promote dental implant (Camlog, Winsheim, Germany) with 3.8 mm in diameter and 13 mm in length were created based on imported Camlog CAD data [23, 23]. Detailed dimensions just as exact internal housing as well as external thread configuration were included in the detailed modelling process. In addition, the construction of corresponding abutments was performed based on imported Camlog CAD data dimensions of 13 mm in height and 3.8 mm in diameter.

Two implant configurations with two and four interforaminal implants were selected for the simulation. The anterior implant placement in the mandible was selected to be identical for both implant configurations. The horizontal implant position at the lateral incisor region was chosen for the 2-implant based configuration as well as for the anterior implants for the four-implant based configuration. Both anterior implants were placed enossally with an implant distance of 13 mm [36]. The 4IF configuration additionally included two posterior implants, which were placed in parallel to the anterior implants in the region of the first premolar about 5 mm mesial to the mental foramen [36]. The lateral implants were placed with a constant distance of 12.5 mm to the anterior implants on both sides [36]. Crestal implant placement was chosen for all implants as horizontal positioning.

The models with two and four incorporated interforaminal implants were then duplicated. Subsequently designed models of a two- and four-implant connecting suprastrucuture, representing a configuration corresponding to a fixed titanium framework similar to a bar or a fixed implant‐prosthodontic reconstruction were added to each corresponding model [37, 38]. Both implant-configuring splinting devices were constructed with identical dimensions in the area between the two anterior implants in terms of design and material thickness.

The combination of all corresponding solid models was conducted in Inventor™ software® (Autodesk GmbH) using Boolean operation method (addition and subtraction) [34, 35, 39]. The experimental study design then included four different models with two implant configurations: model 4IF-U: edentulous mandible with four unsplinted interforaminal implants; model 4IF-S: edentulous mandible with four splinted interforaminal implants; model 2IF-U, edentulous mandible with two unsplinted interforaminal implants and model 2IF-S, edentulous mandible with two splinted interforaminal implants (Fig. 1).

### FEM modelling

All of the resulting CAD models (4IF-U,4IF-S,2IF-U,2IF-S) were entered into the finite element method Simulation section of Inventor software (Autodesk Inventor, Autodesk, San Rafael, USA). Then cross-linking in three dimensions was performed to build corresponding finite element method models. FEA represents an established mathematical technique that enables the reduction of complex geometries into a finite number of voxels (elements), each with a simple geometry. The element format used for the performed cross-linking was selected by the software as parabolic tetrahedrons with four nodes at each corner and one node in the center. The numbers of tetrahedrons and noduli of the four models are presented in Table 1 [34, 35, 39].

**Table 1:** The numbers of tetrahedrons and noduli of the 4 models

Model	Noduli	Elements
2IF-U	953879	638096
2IF-S	958736	639108
4IF-U	1761413	1182135
4IF-S	1790785	1197599

*2IF-U* edentulous mandible model with two unsplinted interforaminal implants, *2IF-S* edentulous mandible model with two splinted interforaminal implants, *4IF-U* edentulous mandible model with four unsplinted interforaminal implants, *4IF-S* edentulous mandible model with four splinted interforaminal implants

All individual structures of the FEM models were defined by specific material properties which are determined as standard values described in the current literature. In addition, the included materials were characterized as isotopic and elastic structures, respectively [40]. The ascribed values are presented in Table 2 [20, 21, 39, 41, 42]. The material properties of a titanium alloy (Ti-6Al-4V) were chosen for the implants and for both the abutments and the superstructure, as reported in previous FEA and clinical studies [43–45].

**Table 2:** Elastic modulus and Poisson ratio of the study materials [21, 31–33]

Materials	Elastic modulus (E) MPa	Poisson ratio
Cortical bone	13.700	0.33
Cancellous bone	1370	0.3
Titanium alloy	110.000	0.34
(Ti-6Al-4V)		

**Fig. 2:**
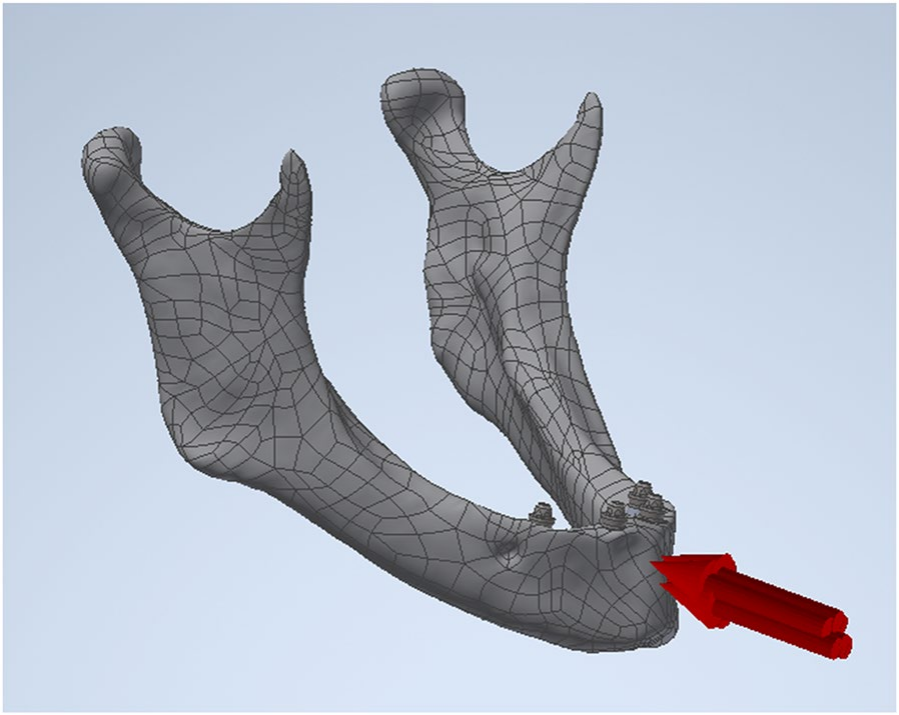
Simulation of frontal symphyseal trauma by application of 1000N.

For the simulation a traumatic load of 1000 N was applied in perpendicular direction to the cortical bone surface of the symphysis (Fig. 2) [20, 21, 23]. A constrainment of the mandible was performed in the proximal portion of the condyles regarding the prevention of free movement in the *x*‐, *y*‐, and *z*‐axes during traumatic loading for simulating the presence of masticatory muscles during trauma [20, 21, 32, 33]. The contact conditions between the single model units of implants, abutments, and suprastructure were specified as constrained [20, 21]. The bone tissue/implant interfaces were considered to be fully osseointegrated [20, 21, 39, 46]. The simulation conditions regarding the force load and application as well as the boundary and contact conditions were identical for all models.

### Strain measurement

**Fig.3:**
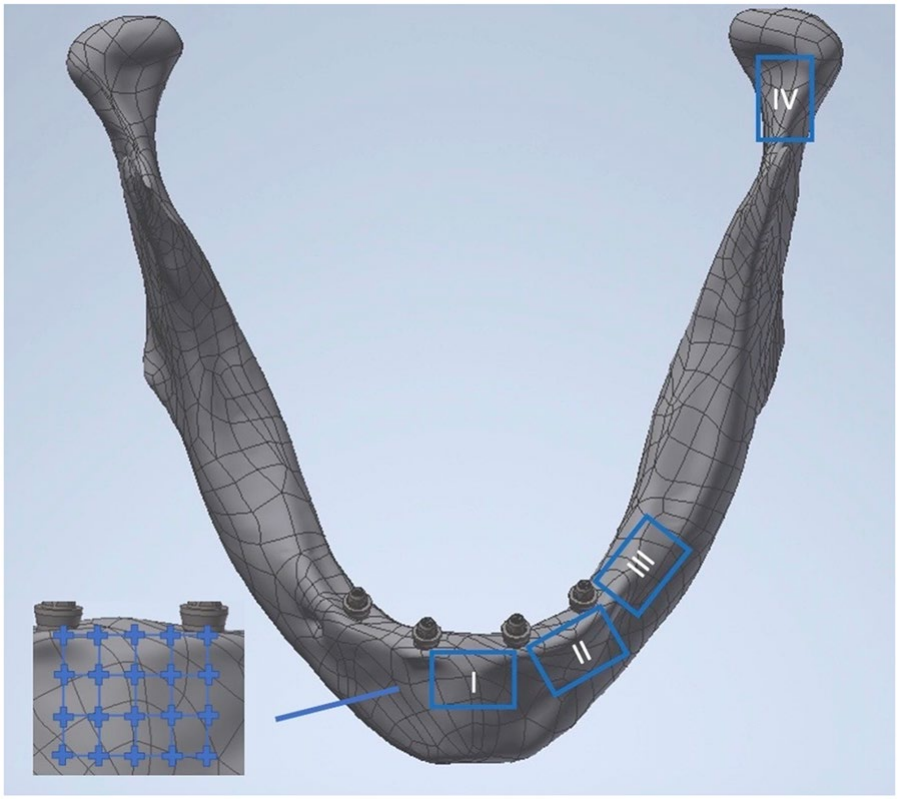
Presents the analysed regions of interest (ROI) evaluated for von Mises stress values. (ROI 1 = anterior mandible; ROI 2 = preforaminal area, ROI 3 = mental foraminal region; ROI 4 = condylar neck region; homogenous distribution of the 20 pre-defined measurement points identical for all ROIs).

The traumatic cortical stress evaluation was conducted in detail for four defined specific regions which were selected on the basis of important areas of the mandible involved in traumatic fractures in recent literature [20, 21, 33]. The evaluated sites which were selected to be identical for all models, were defined as regions of interest (ROI) and were located as follows (Fig. 3):

1.  ROI 1: region between the anterior implants, mandibular body
2.  ROI 2: region between the anterior and lateral implants, preforaminal area
3. ROI 3: region posterior to the lateral implants, in the mental foramen area.
4. ROI 4: region at the condylar neck area.

All regions showed a homogeneous area dimension of 10 × 6.5 mm, and the effective stress calculation in ROIs was conducted at 20 homogeneously distributed pre-defined measurement points at specific superficial cortical mandibular areas [23]. The measurement conditions represented identical inter-point distances, allocation and number of measured control points for all region of interest in all four models (Fig. 3). Therefore, an identical stress calculation of all trauma simulations could be achieved in all models. The traumatic stress evaluation was performed at these predicational survey areas according to von Mises equivalent stress dispersal.

### Statistical analysis

The parameters (von Mises voltage values) of ROIs 1, 2,3 as well as 4 and models 2IF-U, 2IF-S,4IF-U and 4IF-S were tabulated as mean standard deviation. For the comparison of normally distributed continuous variables within each region, repeated analysis of variance or—in the case of non-normality (verification with the Kolmogorov- Smirnov test with Lilliefors correction)—Friedman rank analysis of variance was used. For post-hoc comparisons, Bonferroni-adjusted paired t-tests or Conover post-hoc tests were used. Type I error was set at 5% (2-sided) without adjustment for multiple testing, except for post-hoc comparisons, and P < 0.05 was considered statistically significant. Interindividual comparisons (ROI 1, 2, 3, or 4 for model 2IF-U vs ROI 1, 2, 3, or 4 for model 2IF-S vs ROI 1, 2, 3, or 4 for model 4IF-U, ROI 1, 2, 3, or 4 for model 4IF-S) were performed. For statistical analysis, the statistical software R (version 3.5.2; R Foundation for Statistical Computing, Vienna, Austria; http://www.R-project.org) was used.

## Results

**Fig. 4:**
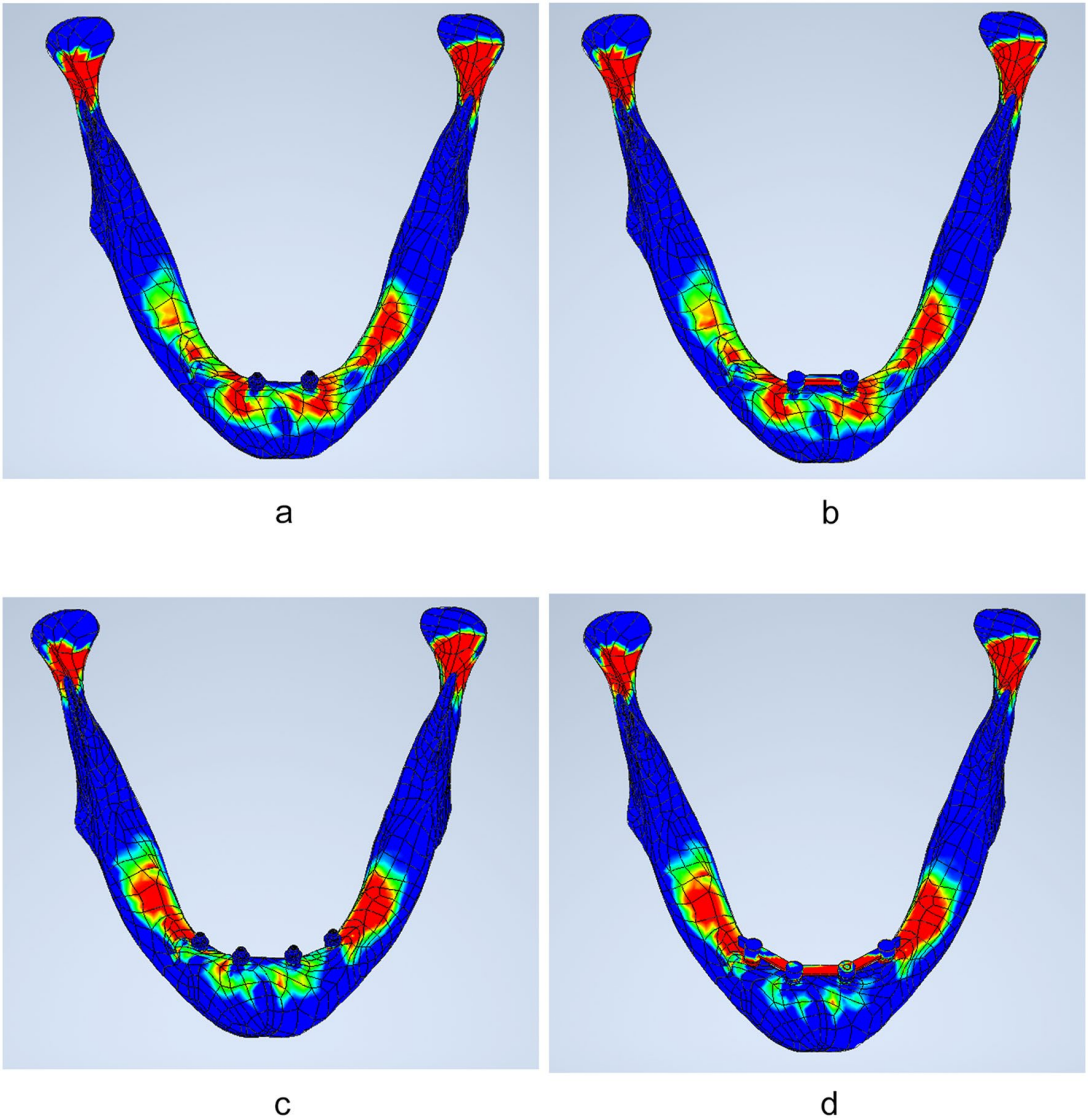
Show the Finite element stress values (von-Mises stress) for the 2IF-U model (**a**), 2IF-S model (**b**), 4IF-U model (**c**) as well for the 4IF-S model(**d**) exposed to symphyseal trauma. *2IF-U* edentulous mandible model with two unsplinted interforaminal implants, *2IF-S* edentulous mandible model with two splinted interforaminal implants, *4IF-U* edentulous mandible model with four unsplinted interforaminal implants, *4IF-S* edentulous mandible model with four splinted interforaminal implants

Figure 4 presents the individual finite element stress values (von Mises stress) evaluated for edentulous mandibular models with four different implant configurations (Fig. 4a: 2-unsplinted interforaminal implants [2IF-U], Fig. 4b: 2-splinted interforaminal implants [2 IF-S], Fig. 4c: 4-unsplinted interforaminal implants [4IF-U], Fig. 4d: 4-splinted interforaminal implant [4IF-S]) exposed to frontal symphyseal application of 1MPa of traumatic stress. The detailed data of the stress values generated for all models as well as for all regions of interest defined (ROI 1 [mandibular symphysis], ROI 2 [preforaminal area], ROI 3 [regio mentalis], ROI 4 [condylar neck) are presented in Table 3 (mean ± SD).

**Table 3:** Detailed stress values (MPa) for all models and regions of interest expressed as mean and standard error values

Configuration	ROI I	ROI II	ROI III	ROI IV
2IF-U:	48.5 ± 11.8	38.8 ± 11.9	45.8 ± 16.7	91 ± 60.2
2IF-S:	46.9 ± 11.7	38.5 ± 11.4	45.1 ± 16.4	91.5 ± 59.4
4IF-U:	42.8 ± 10.7	33.2 ± 8.7	55.9 ± 17.9	70.4 ± 46.4
4IF-S:	31.5 ± 9.9	29.7 ± 6.1	54.1 ± 18.1	69.5 ± 46.3
Comparison: P-value				
4IF-S vs 4IF-U	**0.001**	0.129	0.764	0.996
4IF-S vs 2IF-S	**0.001**	**0.001**	**0.001**	**0.001**
4IF-S vs 2IF-U	**0.001**	**0.001**	**0.001**	**0.001**
4IF-U vs 2IF-S	**0.043**	**0.012**	**0.001**	**0.001**
4IF-U vs 2IF U	**0.001**	**0.03**	**0.001**	**0.001**
2IF-S vs 2IF U	0.857	> 0.999	> 0.999	> 0.999

*2IF-U* edentulous mandible model with two unsplinted interforaminal implants, *2IF-S* edentulous mandible model with two splinted interforaminal implants, *4IF-U* edentulous mandible model with four unsplinted interforaminal implants, *4IF-S* edentulous mandible model with four splinted interforaminal implants

**Fig. 5:**
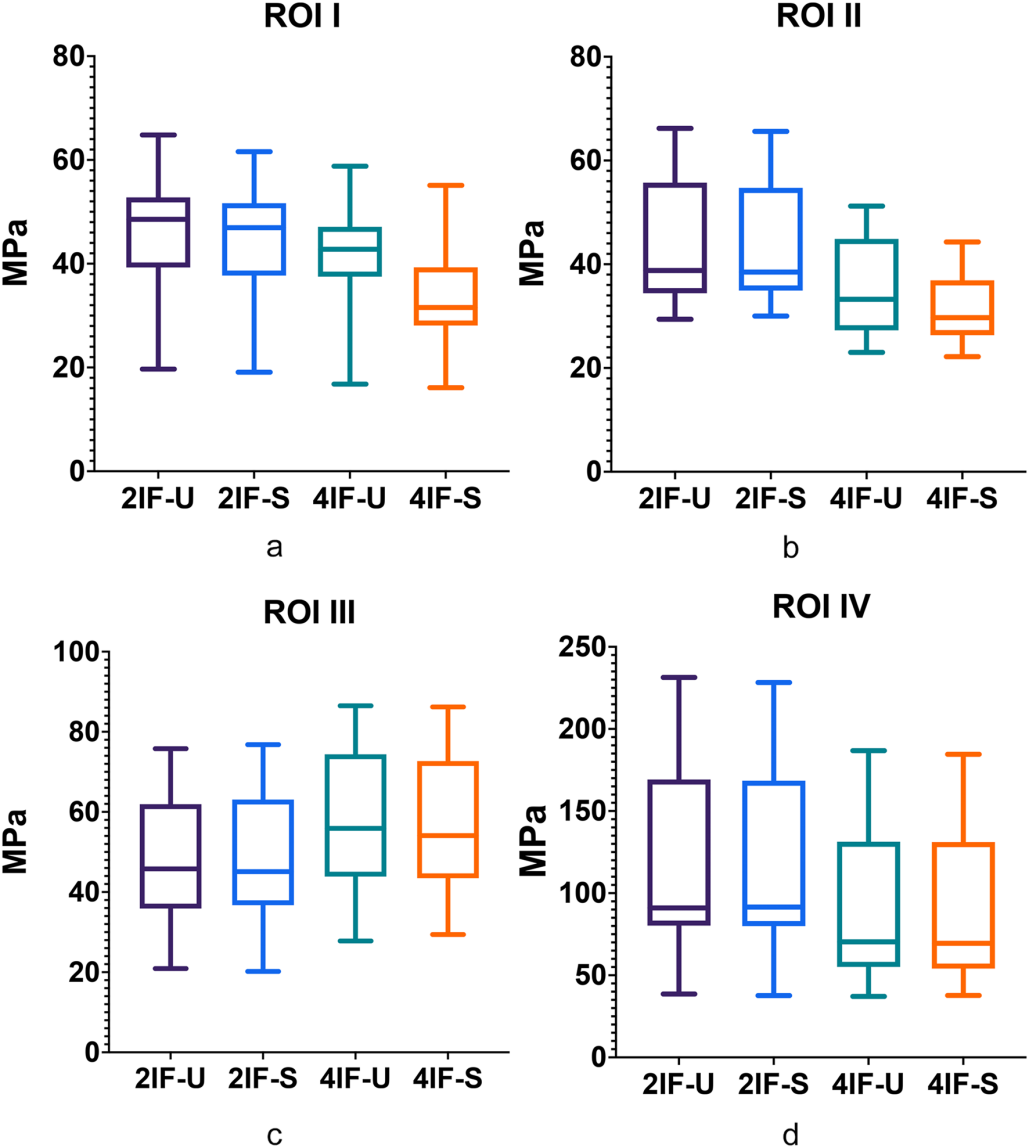
**a-d** Intermodel comparisons of stress values for ROI 1, ROI 2, ROI 3 and ROI 4 for 2IF-U model (**a**), 2IF-S (**b**), 4IF-U (**c**) and 4IF-S (**d**) with simulated frontal trauma. *2IF-U* edentulous mandible model with two unsplinted interforaminal implants, *2IF-S* edentulous mandible model with two splinted interforaminal implants, *4IF-U* edentulous mandible model with four unsplinted interforaminal implants, *4IF-S* edentulous mandible model with four splinted interforaminal implants

Figure 5a-d presents the stress values evaluated expressed as box plots for all ROIs 1—4 enabling intermodel comparisons. For the frontal symphyseal region of interest (ROI I) both models with four implants (4IF-U and 4IF-S) presented significantly (**P < 0.001; P 0.043**) reduced stress values as compared to models with two-implant-supported configurations (2IF-U and 2IF-S). In addition, the model with four splinted interforaminal implants (4IF-S: van Misses stress: 31.5± 9.9 MPa) also represented a significantly reduced stress level versus the edentulous mandible with four unsplinted implants (4IF-U: Van Misses stress: 42.8± 10.7 MPa; **P < 0.001**). In contrast, the stress values evaluated for the mandible with two splinted implants (2IF-S: Van Misses stress: 46.9± 11.7 MPa) and two unsplinted implants (2IF-U: van Misses stress: 48.5± 11.8 MPa) did not differ significantly (P 0.857, Table 3).

Van Misses stress values evaluated for ROI 2 (premental area) are presented in Fig. 5b. In ROI 2, both four-implant models (4IF-U, 4IF-S) demonstrated significantly lower stress values (**P < 0.001**; **0.012,** **0,03**) than both two-implant based configurations (2IF-U and 2IF-S). No significant differences were noted for comparisons within both 4-implant restored models (4IF-U vs 4IF-S; P > 0.129) as well for both two-implant models (2IF-U vs 2IF-S; P > 0.999, Table 3).

Figure 5c presents detailed von Mises stress values (box plots) of the ROI 3 corresponding to the mental foramen area. In the mental foraminal area (ROI 3) the stress values evaluated showed significantly (**P < 0.001**) lower values for both two-implant models (2IF-U, 2IF-S) as compared to both four-implant supported configurations (4IF-U, 4IF-U). However, the stress values in ROI 3 did not differ either within the four-implant or within the two-implant configuration (4IF-U vs 4IF-S P > 0.764 ; 2IF-U vs 2IF-S P = 0.999, Table 3).

By comparing all ROI within each model, the condylar neck area consistently represented the highest stress pattern (Fig. 5d, Table3). For the evaluated condylar neck area at ROI IV both four-implant restored mandibles showed significantly (P < 0.001) lower stress values than both two-implant configurations. However, the stress values in ROI 3 did not differ between the unsplinted and splinted implant models (4IF-U vs 4IF-S, P > 0 0.996; 2IF-U vs 2IF-S P = 0.999; Table3, Fig. 4c,d, Fig.5).

## Discussion

According to the findings of numerous previous studies interforaminal implant placement are reported to have a weakening influence to the atrophic edentulous mandible [20, 21, 23, 47]. Different biomechanical studies could demonstrate that in case of facial trauma an osseointegrated dental implant leads to higher stress distribution and therefore a higher risk of fracture [20, 21]. Kan et al. study results show that the fracture risk increases with increasing inter-implant distance [21]. Additionally, Ayali et al. could show in their study that in case of traumatic forces higher stress levels occur where implants directly come in contact with cortical bone and subsequently a reduction of the risk of bone fracture in the mandible can be achieved by the insertion of the implant into spongious bone monocortically [20].

In addition splinted implant configuration have also been demonstrated providing reduced and beneficial stress conditions under traumatic loading [23, 23, 48, 49]. Therefore, by reducing the implant-surgical risk factors and using the prosthodontic beneficial splinted configuration it was initially hypothesized that an edentulous mandible using two splinted implants show reduced stress exposure and a lower fracture risk than four interforminal implants.

However, the according to the findings of the present experimental study this hypothesis had to be rejected. In particular, it could be shown that in a simulation of a frontal trauma the configurations with two interforaminal implants resulted in higher stress values in the anterior median mandibular area than those in models with four implants (4IF-S, 4IF-U). Regardless of whether the two interforaminal implants were used in splinted or unsplinted configuration, a frontal trauma consistently resulted in increased stress values in the area of the anterior implants and there in the periimplant cortical region, which may be attributed to the weakening of the bone by the implant insertion [20, 21, 23]. Thus, the results of the present experimental study confirm the data of Kan et al. [21] and Ayali und Bilginaylar [20] reporting that with two interforaminal implants exposure to a frontal trauma will result in increased stress values in the area of the periimplant bone as well as in the area of the implants [20, 21, 23, 24].

However, interestingly it could also be noted that in the case of a traumatic force exposure it is especially the number and the regional localization of the implants that show a significant impact on the stress distribution in the area of the anterior mandible [20, 21]. In obvious contrast to two interforaminal implants where the traumatic energy potential is immediately transmitted to the peri-implant cortical bone, exposure to a frontal traumatic force of a configuration with four implants will result in a more even distribution of the stress pattern. In particular, the comparison of four splinted interforaminal implants versus four unsplinted implants showed that the stress values were even more significantly reduced and thus associated with a favorable fracture risk in the symphysis area. This may be attributed to the fact that in the four-implant model the splinting suprastructure provides for transmission of the stress values to the splinted bar and/or the attachments and not directly to the peri-implant cortical bone as in the unsplinted model [23, 24, 50].

However, in obvious contrast to four splinted interforaminal implants the splinting device for two implants shows no significant effect on the stress and fracture behavior. This might be attributed to the reduced volume of splinting (bar length) with subsequently reduced potential of stress absorption as well as to the reduced number of implants (two vs four implants) and, consequently also to the reduced potential of stress distribution [51].

Implant splinting might be compared with the effect of a fixation providing for a positive effect on bone stress values and on fracture risk [52]. It is well known that external pin fixation represents a conventional method for stabilization of fracture segments and will also be used in certain settings in traumatology [52, 53]. A prosthetic bar or the supporting suprastructure for a fixed denture on four implants thus also represents a suitable external splinting even without an original intention and shows the potential of reducing the fracture risk in the symphysis region [23, 24].

In addition, it can be noted that the cortical stress in the preforaminal area (ROI 2) in the models with two inserted implants showed significantly higher values than in four-implant restored models. This may be explained by the fact that in a setting of anterior implant insertion and frontal trauma application the interaction of acceleration, mass inertia and deformation changes of the jawbone must be considered for increased cortical stress conditions [20, 21, 23].

In this respect, each configuration shows an increased stress pattern transmission into the distal region of the posterior implants [23, 24]. This theory of stress pattern distribution to the respective distal area of the posterior implants is also confirmed by the increased stress values in the ROI 2 with the configuration of two implants as well as by increased stress values in ROI 3 in the configuration of four implants. Strikingly, however, an additional splinting—in both implant configurations—produced no significant difference in the evaluated stress values for both the preforaminal area (ROI 2) and for the area around the mental foramen (ROI 3) concluding no influence of the suprastructure on the stress conduction into the posterior area [23, 24].

Moreover, the results of the present FEA show that upon frontal force application (symphyseal) the highest stress values—and consequently also the highest fracture risk—were invariably seen in the area of the condylar neck in all of the models [23, 24]. This obviously confirms the results of De Santos [33] for the edentulous mandible without implants and of Bilingylar and Ayali [20] for implant-treated mandibular models. According to Schwartz-Dabney and Dechow the bone relationships in the mandibular neck are narrower so that this region shows a lower bone stability which may consequently lead to an increased fracture tendency [54].

However, as a complementary finding the FEA analysis shows that the stress pattern in the condylar neck (ROI 4) in the models with two implants inserted was significantly increased versus the models with four implants. While the splinting configuration did not show any significant difference within both models. As the force load with a frontal trauma in the implant-treated mandible will be absorbed in the implant-adjacent bone regions, a reduction of the number of implants will consequently only result in a decreased reduction in the area of the condylar neck [23, 24]. Duplicating the number of implants from two to four will not only provide for stress absorption in an available implant connection (splint), but also in the peri-implant bone and will thus result in a significant stress reduction in the condylar neck and consequently provide for a reduced fracture risk in the condylar neck [23, 24].

## Summary

Considering the results for all implant configurations and mandibular regions analyzed, the study was able to demonstrate that the configuration of four splinted implants provides for the most favorable stress conditions upon exposure with traumatic frontal force. This configuration shows a reduced stress distribution not only in the condylar neck, but also in the area of the symphysis and thus provides for a reduced fracture risk [21, 23, 24, 55]. Although an increased stress behavior and an increased fracture risk could be noted in the mental region with four interforaminal implants (splinted or unsplinted), this fracture region can be considered as a rather favorable site with respect to the surgical treatment options as compared to a fracture in the collum [56, 57].

Under adequate consideration of the limitations of this study, it must be noted that this study had an experimental design and only presents the changes on the objects studied [20, 21, 23]. Certainly, the risk of a mandibular fracture must be evaluated by varying degrees of mandibular atrophy and according to the bone quality of the mandible [33, 58]. The study served for exploring experimental findings which are to show a change in the fracture pattern and a relocation of potential injuries to sites providing for improved surgical access and/or facilitated treatment procedures [20, 23, 33].

Although recent literature reports have documented FEA in the mandible under traumatic conditions as reliable and accurate non-invasive method for evaluating biomechanical behavior and the anterior mandible as implantation site in our study has shown a homogenous structure, the results of this must currently be interpreted with appropriate caution [20, 21, 23, 55].

## Data Availability

Not applicable.
