# Correction: The effect of frontal trauma on the edentulous mandible with four different interforaminal implant-prosthodontic anchoring configurations. A 3D finite element analysis

**DOI:** 10.1186/s40001-024-01697-8

**Published:** 2024-02-21

**Authors:** Stefan Krennmair, Michael Malek, Raphael Stehrer, Philip Stähler, Sven Otto, Lukas Postl

**Affiliations:** 1 Medical Faculty, Johannes Kepler University Linz, Altenberger Strasse 69, 4040 Linz, Austria; 2 Department of Oral and Maxillofacial Surgery, Kepler University Hospital, Johannes Kepler University, Krankenhausstrasse 7a, Linz, Austria; 3 NumBioLab, Ludwig-Maximilians University of Munich, Munich, Germany; 4 Department of Oral and Maxillofacial Surgery, Ludwig-Maximilians-University Munich, Lindwurmstrasse 2a, 80337 Munich, Germany

**Correction: European Journal of Medical Research (2023) 28:608 ** 10.1186/s40001-023-01580-y

In the original version of this article [1], the given and family names of all the authors except "Philip Stähler" were swapped incorrectly. The corrected given and family names of the authors should read as "Stefan Krennmair, Michael Malek, Raphael Stehrer, Philip Stähler, Sven Otto and Lukas Postl". instead of "Krennmair Stefan, Malek Michael, Stehrer Raphael, Philip Stähler, Otto Sven and Postl Lukas". The original article has been corrected.
